# Signatures of competition and strain structure within the major blood‐stage antigen of *Plasmodium falciparum* in a local community in Ghana

**DOI:** 10.1002/ece3.3803

**Published:** 2018-03-01

**Authors:** Mary M. Rorick, Yael Artzy‐Randrup, Shazia Ruybal‐Pesántez, Kathryn E. Tiedje, Thomas S. Rask, Abraham Oduro, Anita Ghansah, Kwadwo Koram, Karen P. Day, Mercedes Pascual

**Affiliations:** ^1^ Department of Ecology and Evolution University of Chicago Chicago IL USA; ^2^ Department of Biology University of Utah Salt Lake City UT USA; ^3^ Theoretical Ecology Group Institute for Biodiversity and Ecosystem Dynamics University of Amsterdam Amsterdam The Netherlands; ^4^ School of Biosciences Bio21 Institute The University of Melbourne Melbourne Vic. Australia; ^5^ Department of Microbiology New York University New York NY USA; ^6^ Navrongo Health Research Center Navrongo Ghana; ^7^ Noguchi Memorial Institute for Medical Research University of Ghana Legon Ghana; ^8^ The Santa Fe Institute Santa Fe NM USA

**Keywords:** antigenic diversity, modularity, niche partitioning, *Pf*EMP1, strain theory, *var* gene

## Abstract

The concept of niche partitioning has received considerable theoretical attention at the interface of ecology and evolution of infectious diseases. Strain theory postulates that pathogen populations can be structured into distinct nonoverlapping strains by frequency‐dependent selection in response to intraspecific competition for host immune space. The malaria parasite *Plasmodium falciparum* presents an opportunity to investigate this phenomenon in nature, under conditions of high recombination rate and extensive antigenic diversity. The parasite's major blood‐stage antigen, *Pf* EMP1, is encoded by the hyperdiverse *var* genes. With a dataset that includes thousands of *var* DBLα sequence types sampled from asymptomatic cases within an area of high endemicity in Ghana, we address how *var* diversity is distributed within isolates and compare this to the distribution of microsatellite allelic diversity within isolates to test whether antigenic and neutral regions of the genome are structured differently. With respect to *var* DBLα sequence types, we find that on average isolates exhibit significantly lower overlap than expected randomly, but that there also exists frequent pairs of isolates that are highly related. Furthermore, the linkage network of *var* DBLα sequence types reveals a pattern of nonrandom modularity unique to these antigenic genes, and we find that modules of highly linked DBLα types are not explainable by neutral forces related to *var* recombination constraints, microsatellite diversity, sampling location, host age, or multiplicity of infection. These findings of reduced overlap and modularity among the *var* antigenic genes are consistent with a role for immune selection as proposed by strain theory. Identifying the evolutionary and ecological dynamics that are responsible for the nonrandom structure in *P. falciparum* antigenic diversity is important for designing effective intervention in endemic areas.

## 1 INTRODUCTION

The ecological concept of niche partitioning is present in the theory of strain diversity at the interface of ecology and evolution of infectious disease dynamics. Mathematical and computational models have shown how strain structure can emerge from intraspecific competition for hosts mediated by specific immunity (Artzy‐Randrup et al., 2012; Buckee, Koelle, Mustard, & Gupta, 2004; Gupta, Ferguson, & Anderson, 1998). One dynamical outcome of such frequency‐dependent competition is the coexistence of strains with limited antigenic overlap, despite frequent outcrossing through recombination or reassortment (e.g., Artzy‐Randrup et al., 2012; Buckee et al., 2004; van Noort, Nunes, Weedall, Hviid, & Gomes, 2010). Despite different organization levels, there is conceptual overlap between strain theory and ideas in community ecology on limiting similarity and non‐neutral, stabilizing forces that allow species coexistence within communities (e.g., Cavender‐Bares, Kozak, Fine, & Kembel, 2009; Chesson, 2000). In the context of infectious disease dynamics, trait variation concerns molecular variation in major antigens, and limiting similarity would be reflected as linkage disequilibrium between antigenic alleles that is not explainable by physical linkage or other recombination constraints, geographical population structure due to geography or host heterogeneity, or a general lack of sexual recombination (i.e., clonality).

*Plasmodium falciparum* presents a challenge and an opportunity to investigate this phenomenon in nature from the perspective of the hyperdiverse multicopy *var* gene family, which encodes the major antigen of the parasite's blood stage—*P. falciparum* erythrocyte membrane protein 1 (*Pf*EMP1). Because *Pf*EMP1 is the primary target of naturally acquired immunity to *falciparum* infection (Chan et al., 2012), we expect *var* gene diversity to be the playing field for parasite competition over host immune space. Thousands of distinct *var* types circulate together in host communities of highly endemic regions (Chen et al., 2011; Day et al., 2017). It is thought that this diversity has been generated through rapid recombination within the gene family and maintained through thousands of years of evolution through strong balancing selection (Zilversmit et al., 2013). It is also estimated that host individuals in highly endemic areas are infected with hundreds of distinct strains of *P. falciparum* malaria over the course of their lives. It is poorly understood whether and how these gene variants are organized into genomic repertoires at the population level (Day et al., 2017). See the Information Box below for a more in‐depth discussion of *var* gene biology and *P. falciparum* malaria.

While phylodynamic approaches have yielded valuable insights into strain variation in HIV and influenza, different theoretical and empirical approaches are essential for studying the strain dynamics of *P. falciparum* due to the highly recombining nature of this pathogen, and its reliance on diverse multicopy antigenic genes. These attributes of *P. falciparum* also mean that partial discordance among strains may be the expectation (Artzy‐Randrup et al., 2012) rather than the complete lack of overlap of earlier models. Furthermore, we might expect a pattern where isolates are either highly related due to transmission dynamics or highly dissimilar due to niche partitioning.

A recent analysis of a sample from all children in a highly endemic population in Bakoumba, Gabon, has revealed a nonrandom population structure of the parasite with limited overlap between isolates (Day et al., 2017). Here, we extend the study of population structure by sampling the asymptomatic reservoir in *both* adults and children within a local community of high *P. falciparum* endemicity in Ghana. Our dataset includes thousands of natural antigenic variants, representing exceptionally detailed genetic information underlying “trait” variation within a competitive system. We analyze the structure of *P. falciparum var* sequence diversity in this community to determine whether it supports the hypothesis of strain structure emerging from immune selection against recombinants. Specifically, we consider how *var* antigenic diversity is distributed in isolates and compare patterns of *var* type diversity to patterns of microsatellite allele diversity in order to test whether selection is shaping *var* diversity in fundamentally different ways than putatively neutral regions of the genome. We investigate two elements of structure fundamental to predictions of strain theory: a limited overlap between *var* gene repertoires and a high linkage between pairs of *var* genes.

While an understanding of strain structure in *P. falciparum* has implications for malaria intervention design and eradication programs, it also presents broader connections to the interface of disease ecology and population genetics. Evidence for niche partitioning in this system could have implications for theory and field work on other competitive systems undergoing evolutionary change, particularly those that are similar to *P. falciparum* in the reliance on multicopy antigenic gene families and/or high rates of recombination (Deitsch, Lukehart, & Stringer, 2009).

## 2 MATERIALS AND METHODS

### 2.1 Study site and sampling

To evaluate the reservoir of *Plasmodium* spp. in Bongo District (BD), located in the Upper East Region (UER) of Ghana, an age‐stratified cross‐sectional study was carried out at the end of the dry season in June 2012. BD is characterized by marked seasonal transmission, and malaria represents a major public health concern for the district. Details on the study design, study population, and data collection procedures have been described previously (Ruybal‐Pesantez et al., 2017). Briefly, sampling was carried out across two broad catchment areas (Vea/Gowrie and Soe) in BD that were selected because they were considered to be similar in population size, age structure, and ethnic composition. It was however hypothesized that they may differ with respects to malaria transmission intensity and seasonality as Vea/Gowrie is proximal to the Vea dam/irrigation area, while Soe is not near any large bodies of water, although smaller dams for irrigation are scattered throughout the area. The catchment areas were further divided into smaller villages: Vea, Gowrie, Soe Sanabisi, and Soe Boko, with participants enrolled from sections within these villages (Vea: Gonga and Nayire; Gowrie: Nayire Kura and Tingre; Soe Sanabisi: Tindingo and Akulgoo; and Soe Boko: Tamolinga and Mission Area). For these analyses, participants with microscopically confirmed *P. falciparum* infections were included (*N* = 267). Methods related to the microscopy, *msp2* PCR, and the microsatellite PCR are described in detail in (Ruybal‐ Pesantez et al., 2017). Microsatellite data are available for 200 *P. falciparum* samples, and *var* DBLα tags were sequenced for 209 *P. falciparum* samples.

### 2.2 Diversity of *var* DBLα types and microsatellite alleles

We analyzed the *var* antigenic diversity within 209 asymptomatic individuals in Ghana with *P. falciparum* infections. For 163 of these individuals, we also sequenced 12 microsatellite loci as described in detail in (Ruybal‐Pesantez et al., 2017). The total number of observed peaks (alleles) at each locus was used to estimate MOI. Single‐clone infections were defined as those with at most one microsatellite allele at every microsatellite locus. For multiple‐clone infections, the dominant peak (allele) at each of the 12 microsatellite loci was determined for each isolate. The dominant peaks made up what we term the “dominant haplotype” or “dominant infection” for each isolate. Isolates that had an MOI of 1 or 2, determined by the maximum number of peaks observed at a given microsatellite loci, made up the so‐called “high confidence infections”, and this represents a standard method used in the field (Schultz et al., 2010). Due to high levels of MOI, we were able to determine high confidence infections for only 59 of the 163 isolates. Considerable sequence diversity was observed within types, both within and between isolates. Because this represents a mixture of natural sequence diversity and sequencing errors, and because these two sources cannot be distinguished using our methods, we ignored within‐type sequence diversity in this study.

### 2.3 *Var* sequencing methods and type assignment

DBLα, the only domain found in nearly all *var* genes, is a molecular marker of *var* gene diversity (Kraemer & Smith, 2003; Lavstsen, Salanti, Jensen, Arnot, & Theander, 2003; Smith, Subramanian, Gamain, Baruch, & Miller, 2000; Taylor, Kyes, Harris, Kriek, & Newbold, 2000). With average read lengths of 400 bp or greater using 454 sequencing, we sequenced the entire length of the PCR amplicon without the need for assembly (Day et al., 2017; Rask, Petersen, Chen, Day, & Pedersen, 2016).

We assigned DBLα sequences to *var* DBLα sequence “types” in a manner consistent with the 96% nucleotide identity definition commonly used (Barry et al., 2007). More specifically, DBLα types are defined here using a clustering algorithm applied to the raw sequence data, such that each DBLα sequence type cluster corresponds roughly to sequences with a >97% amino acid sequence identity. This threshold is consistent with the majority of prior work defining distinct types within DBLα tag sequences because it ensures that each distinct sequence type very likely represents a naturally occurring distinct variant (i.e, and is not merely the result of sequencing errors).

Most analyses were run using Mathematica v8 scripts unless otherwise noted. We translated DNA sequences to AA sequences using the software program EMBOSS Transeq (Goujon et al., 2010; Rice, Longden, & Bleasby, 2000). We excluded from the analysis sequences that had an unexpected reading frame, apparent frameshift substitutions, or stop codons.

Three genomic isolates were used as positive controls for our sequencing and analysis methods: 3D7, Dd2, and HB3. These samples differ from our field isolates in several respects: The number of *var* genes in each genome is known; the MOI is exactly 1; the complete set of DBLα sequences is known with high precision so it is possible to identify multiple *var* sequences of the same type within these genomes.

### 2.4 Measuring relatedness

Relatedness is measured as the number of DBLα types or microsatellite alleles shared between two isolates divided by the average number of DBLα types or microsatellite alleles in an isolate, for that pair. For DBLα types, this is equivalent to pairwise type sharing (PTS) as defined by Barry et al. (2007).

### 2.5 *Var* repertoire overlap indices

One major aspect of population structure we examined is the overlap among pairs of isolates. We compared the overlap among observed isolates to the overlap among randomized isolates using two different indices: pairwise type sharing (PTS) and the Jaccard similarity (JS) index. If isolate A has a repertoire of *n*_A_ types and isolate B has a repertoire of *n*_B_ types, and the two isolates share a total of *n*_AB_ types, and the union of the two sets is *U*_AB_, we define PTS_AB_ = 2*n*_AB_/(*n*_A_ + *n*_B_) and JS_AB_ = *n*_AB_/*U*_AB_. The PTS similarity index, which was designed for comparing *var* repertoires, was defined here exactly as in Barry et al. (2007).

We address whether the observed overlap differs from random by randomizing the *var* DBLα sequence types in isolates to make a null distribution. We take into consideration several aspects of the observed data to construct a null hypothesis. First, in the field isolates, we can never observe multiple copies of the same DBLα type because we do not determine genomic location and any within‐type sequence variation cannot be distinguished from sequencing errors. In our null distribution of randomized isolates, we maintain the binary nature of the type‐isolate matrix so that there are no repeated DBLα types in any randomized isolates. We also preserve the total number of isolates and DBLα types, the number of DBLα types sampled per isolate, and the observed frequency distribution of DBLα types within the dataset. We preserve these aspects of the observed data by maintaining row and column totals of the matrix of DBLα types in isolates while randomizing the 0/1 entries of the matrix. Finally, we also preserve the connectedness of the original matrix during the randomization. We then asked whether the observed distribution of overlap indices between isolates differed from the distributions of overlap indices for these randomizations. We use an efficient switch algorithm to build our null distribution, called the Curveball algorithm (Strona, Nappo, Boccacci, Fattorini, & San‐Miguel‐ayanz, 2014). The method was implemented with a program written for assessing modularity and stability of ecological networks (Grilli, Rogers, & Allesina, 2016). We sampled every 100 swap units, which is four times the recommended minimum in Strona et al. (2014) (each swap unit is equal to the number of rows or columns, whichever is lower, and it only counts the number of actual swaps in the matrix as opposed to all the proposed swaps).

### 2.6 Linkage coefficient and modularity of linkage networks

Another aspect of structure predicted by strain theory is that of linkage between *var* genes. This linkage emerges from selection against recombinants and is maintained dynamically. We used the linkage disequilibrium coefficient, *D*, to measure correlations between DBLα types with respect to their presence in isolates. We created networks of DBLα types that had *D* values above a given threshold of *D* > 0.02 and analyzed the structure of these networks. We carried out linkage analysis using the entire dataset and then repeated the analysis using just singly infected isolates. For the microsatellite alleles linkage network, we also used a threshold of *D* > 0.02.

For the DBLα type linkage network, we only included DBLα types that occur more than once in the dataset as these are the only ones that can have significant linkage relationships. We only considered positive linkage disequilibrium coefficients because negative linkages can result from alleles sharing a locus, and the genomic location of DBLα types is not determined in this study. We considered *D* values statistically significant when they exceeded the threshold described in Hedrick, Jain, and Holden (1978).

To identify sets of *var* genes that tend to co‐occur together, we conducted modularity analysis of *var* linkage networks. For comparison, the modularity of microsatellite linkage networks was also performed. We used the software MODULAR (Marquitti, Guimarães, Pires, & Bittencourt, 2013), which defines the optimal number of modules within a network and specifies their members, the overall modularity of the network, and the significance of the modularity relative to a null model that preserves the original degree distribution. While the method was designed for ecological systems, it is based on general network theory and the definition of modularity as “subsets of tightly connected elements”. The following parameter settings were used to identify modules in both the *var* and microsatellite linkage networks. All linkage disequilibrium coefficients greater than 0.02 (*D* > 0.02) were used to construct a unipartite network, expressed as a binary matrix. We specified that 1,000 randomized matrices should be used to build the null model for determining the significance of the modularity, and we used spectral partitioning as the optimization method. In the case of the DBLα type linkage network, we confirmed significance (*p* < .01) using a more conservative null model than the ones generated by MODULAR. This conservative null was based on 100 randomizations that maintained the row and column totals of the original matrix as described below.

For the DBLα type linkage network, we used the 29 single infection isolates to determine linkage disequilibrium coefficients. For the microsatellite allele linkage network, we used the 55 complete high confidence infections to determine linkage disequilibrium coefficients. To address whether forces related to transmission or demography, that would shape diversity at microsatellite and *var* loci similarly, might be responsible for structure observed in the *var* linkage network, we created Figure 8 using the 45 isolates for which we have both complete microsatellite high confidence infections and DBLα type data (regardless of whether they are single infections by the strict microsatellite criteria).

### 2.7 HB recombination network

An important alternative neutral explanation for the existence of the *var* linkage modules is the so‐called *var* recombination hierarchy, which describes how recombination occurs preferentially among certain groups of *var* genes. We sought to test whether these *var* recombination constraints could explain the linkage modules by first building a *var* recombination network and then testing whether the linkage modules clustered within this network. The recombination network was constructed by identifying homology blocks (HBs), which are conserved units of *var* recombination that are present in all the DBLα tags. HBs were identified using the VARDOM web server (Rask, Hansen, Theander, Pedersen, & Lavstsen, 2010), with a gathering cutoff of 9.97 to define a match. We then connected DBLα types with an edge when they shared a homology block, as this can be considered direct evidence of a historical recombination event between these two types. We did not consider the three HBs that are >50% frequent in the DBLα tag (HB 5, 14, and 36).

### 2.8 Randomizations for the assessment of population structure

We asked whether the number of shared DBLα types and shared microsatellite alleles between areas A and B is more or less than what we would expect randomly given the number of types, and their distributions, in each of the areas. To address this question, we randomized the catchment area location of each of the isolates, so that the number of isolates in each area was conserved, and then we assessed the number of shared DBLα types or microsatellite alleles in the two areas. We assessed the number of shared DBLα types or microsatellite alleles each time we performed the randomization and built a distribution from 10,000 randomizations, which we used to calculate a one‐tailed *p*‐value for the observed number of shared types or microsatellite alleles. For the microsatellite analysis, the sample included all alleles from the dominant infections of the 163 isolates for which we could calculate a dominant microsatellite haplotype and for which we had *var* (and location) data. For the *var* analysis, the sample consisted of all unique DBLα types for which we had sampling location data.

### 2.9 Population genetics variables

While the concept of expected heterozygosity and homozygosity does not clearly pertain to the largely nonallelic *var* gene family, we defined an analogous statistic that is appropriate for *var* genes—*var* expected heterozygosity (*H_v_*)—and we used it here to address questions about *var* gene diversity at different hierarchical levels of the population. Pairwise type sharing (PTS) between isolates is a concept that was introduced by Barry et al. (2007) in part because it adapts useful concepts from population genetics: expected heterozygosity (*H*) and expected homozygosity (1 − *H*), to the case of *var* genes. PTS can be considered roughly equivalent to expected homozygosity. Here, we extended the analogy further for the purpose of randomizations and the construction of *F*_*ST*_‐like statistics, and we introduced the terms “*var* expected heterozygosity” (*H_v_*), “*var* expected homozygosity” (1 − *H_v_*), and *var*−*F*_*ST*_ (*F*_*STv*_) for clarity.

Strictly for the purpose of creating metrics to gauge *var* diversity, we imagined that all DBLα types are alleles at a single locus. While this assumption is excessively simplistic, given the ectopic nature of their recombination system, it is not altogether biologically inappropriate. In this sense, we considered a parasite to be a ~60N individual with respect to *var* genes. The standard method for assessing expected heterozygosity and related statistics is to randomly sample haploid organisms (“gametes”) from the population. In our case, we sampled single *var* genes from the population to represent the gametes. We then combined two of these single *var* gene gametes to create diploid parasites, each containing two *var* genes. We did this in order to describe patterns in the observed diversity that differs between the two catchment areas, or other subsets of our isolate sample. Differences between the DBLα type diversity sampled from different locations were analyzed at a range of resolutions: at the low resolution of the two catchment areas down to the eight sections, and we also considered the DBLα types within versus between distinct isolates located in the same section.

To ask whether expected *var* homozygosity within the Vea/Gowrie or Soe catchment areas is greater than expected, we randomized the existing *var* gene variation into 2N isolates (in order to avoid resampling from the limited set of genes). We reshuffled the genes 10,000 times to create a distribution. We tested whether the microsatellites reflected geospatial structure between the two catchment areas by considering the number of shared microsatellite alleles between the areas and asking whether this number deviated significantly from the random expectation. We also asked whether the catchment areas had a lower number of shared microsatellite alleles than expected at random, using the randomization procedure described above.

## 3 RESULTS

In the complete *var* dataset of 209 isolates, we identified 11,308 unique DBLα sequence types, with 3,022 occurring more than once. Of the 163 isolates for which we have both *var* and microsatellite data, 29 had a single allele at each of the microsatellite loci. We refer to this as our single infection dataset, and in this we identified 1,522 unique DBLα sequence types, with only 125 of these occurring more than once. Six additional isolates have a single microsatellite allele at each locus, but for these we have no *var* data (where we do not require *var* data, we use all 35 microsatellite‐estimated single infection isolates).

**Figure 1:**
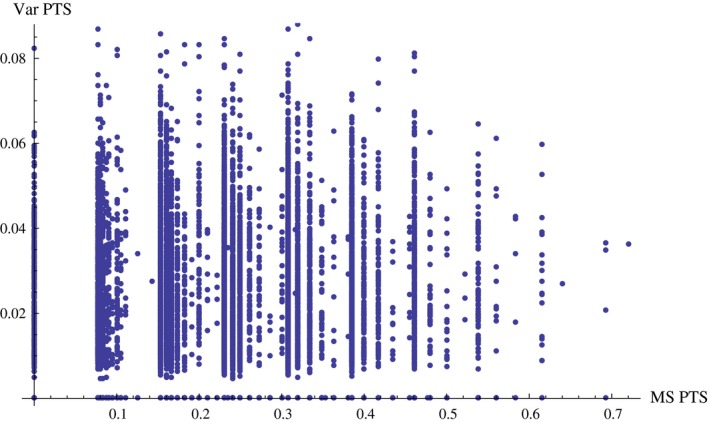
Relatedness of isolates with respect to *vars* versus microsatellites. There is no apparent correlation between these two variables (correlation coefficient = 0.052). Relatedness is measured as the number of DBLα types shared between two isolates divided by the average of DBLα types per isolate in that pair

**Figure 2:**
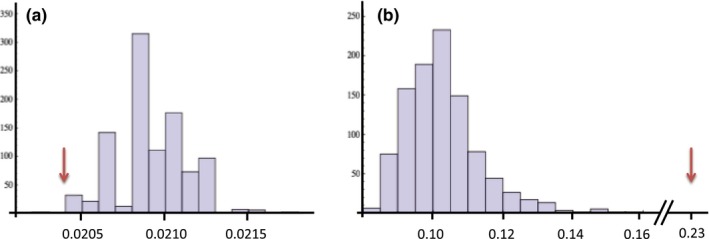
(a) The median Jaccard similarity indices for 1,000 randomizations (maintaining connectedness). Observed median Jaccard similarity (red arrow) is 0.0204, corresponding to a *p*‐value of 0.036. Using randomizations that do not maintain connectedness, *p *=* *0.006. (b) The maximum Jaccard similarity indices for 1,000 randomizations (maintaining connectedness). Observed median Jaccard similarity (red arrow) is 0.228, corresponding to a *p*‐value <0.001

Based on the concept of pairwise type sharing, we found no relationship between isolate relatedness with respect to *var* genes and isolate relatedness with respect to microsatellite alleles. Figure 1 shows the relationship between microsatellite allele relatedness versus DBLα type relatedness for all pairs of isolates for which we have a dominant microsatellite infection and for which we also have *var* data (*N* = 163). Particularly, when we consider the most informative isolates and DBLα types (by first removing DBLα types that occur only once, and then removing the isolates with <20 DBLα types), we find that the median observed Jaccard similarity index is significantly smaller than expected at random (*p *= .036) and that the maximum observed Jaccard similarity index is significantly greater than expected at random (*p *< .001) (Figure 2). We find the same pattern for the pairwise type sharing index, with the same *p*‐values (data not shown). In summary, we find that field isolates are on average (i.e., as reflected by a *median*) less related than if they were random subsets of the *var* diversity, but that simultaneously there also exists some rare pairs of highly related isolates, which are also not expected randomly. (*F*_*STv*_ findings were consistent with the above; data not shown.)

For the *var* dataset (using the 125 DBLα types that occur more than once in the 29 single infections), we find about twice as many positive *D* values exceeding the *p* < .05 significance threshold as expected by chance if there are no truly significant linkages (725 versus expectation of 387). For the microsatellite dataset (using the 118 alleles within the set of 55 isolates for which we have a complete set of 12 alleles in the dominant haplotype), we find slightly more positive *D* values exceeding the *p* < .05 threshold as expected by chance (374 versus expectation of 345). This is a considerably less extreme discrepancy than in the case of the *var* genes.

**Figure 3:**
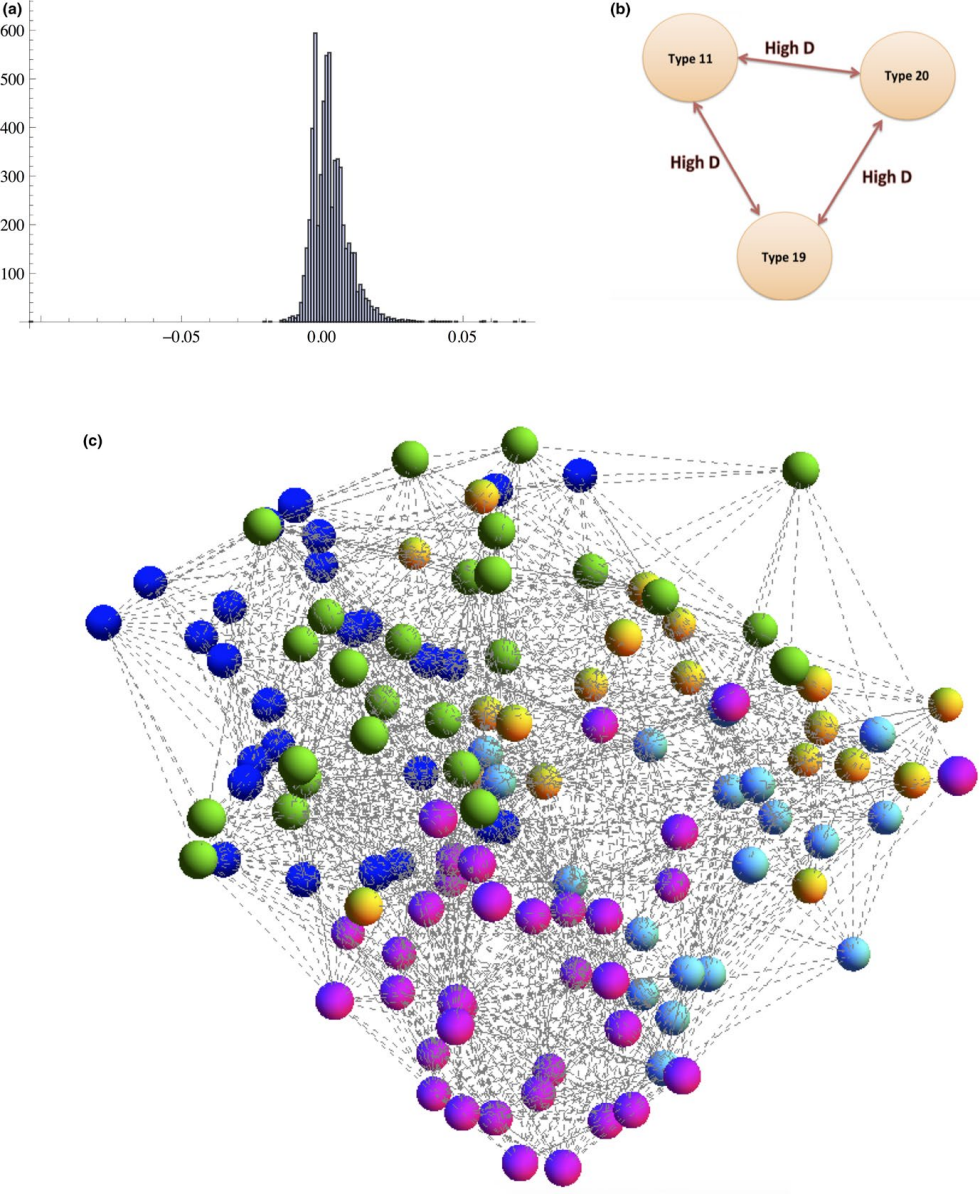
(a) Distribution of *D* values. Note that *D* > 0.02 represents the tail of the distribution. (b) *Var* linkage clique: DBLα types 20, 19, and 11 were found to correlate strongly with one another, (c) Significant modularity in a DBLα type linkage network. *D* values >0.02, only single infections. The different colors of the nodes correspond to the different identified modules. We find significant modularity in this network when compared to randomizations that preserve the degree distribution (*p* < .00001). The colors of the nodes correspond to the colors in Figure 6. We identify five modules, and the modularity is ~0.28. The modularity of the null networks is approximately half this observed value

We next looked for structure among the *var* linkage network: DBLα types that circulate within the host population together more often than expected at random. This analysis does not rely on predefined geographical divisions, but instead is based on considering the DBLα type linkage network. Figure 3c shows the DBLα type linkage network, where edges reflect *D* > 0.02 among the singly infected isolates. We set a threshold of *D* > 0.02 because it reflects the extreme tail end of the *D* distribution (Figure 3a). In all repertoires of DBLα types and in the repertoires of single infection isolates, we find many significant positive correlations and a few DBLα types that are very strongly correlated (Figure 3b). These very strong correlations reflect the existence of one very strong clique of DBLα types that tend to appear together with high frequency: type 20, type 19, and type 11. The linkage coefficients among these three DBLα types are at the highest end of the distribution for this population. We examined whether transmission dynamics could explain its existence by considering the microsatellite relatedness of isolates containing this clique. Interestingly, the microsatellite relatedness of the isolates in which we find this clique is lower than average (0.19 compared to a mean of 0.24). This implies that there is no high “background” genome relatedness among the isolates that contain this clique, so a finite population size (i.e., inbreeding, simple transmission dynamics, or host population structure) cannot explain its existence. The frequency at which this clique occurs (15 times in 209 isolates) is much greater than the random expectation given the independent frequencies of the types (which is approximately 16 times per 10,000 isolates).

We also applied a module‐detecting algorithm (the software program MODULAR) to the DBLα type linkage network and demonstrated that this network is significantly modular (*p* ≪ .001). We then tested different possible causal scenarios for these modules. We found that the *var* linkage network is optimally decomposed into five distinct modules (Figure 3c). We then analyzed whether these modules correspond to several potential causal variables: *var* recombination groups, sampling location, host age, host MOI, or any forces also shaping microsatellite linkage modules. In other words, we ask whether forces other than immune selection can explain the modular pattern of *var* gene linkage.

**Figure 4:**
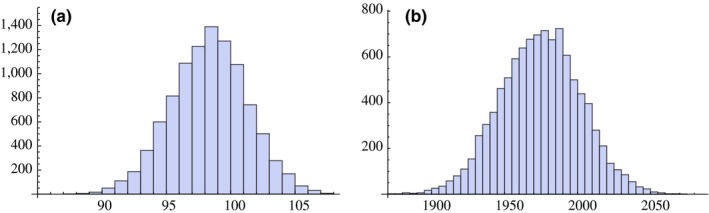
(a) Random expectation for the number of shared microsatellite alleles based on 10,000 randomizations. Observed value is 98, corresponding to a *p*‐value of 0.59. (b) Random expectation for the number of shared *var* DBLα types based on 10,000 randomizations, between the two catchment areas. Observed value is 1915, which corresponds to a *p*‐value of 0.0179

**Figure 5:**
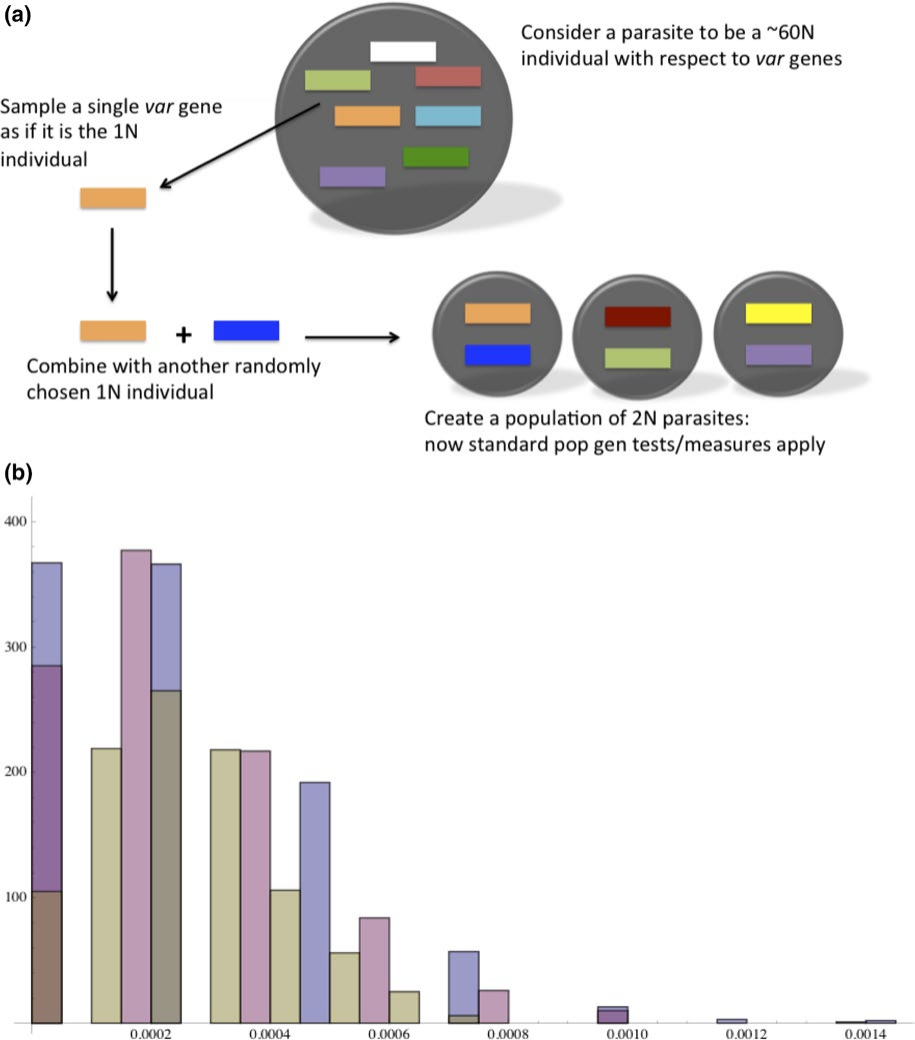
(a) Population genetic statistics for *var* genes. Because haplotype‐based analysis is not possible for *var* genes in a high MOI setting, here we imagine all *var* genes sharing a single locus and then perform *F*_*ST*_ and *H*‐based tests. (b) The expected homozygosity (1 − *H_v_*) distribution within Vea/Gowrie (blue), within Soe (pink), and within the combined population (yellow)

First, we addressed the variable of sampling location by explicitly testing for geospatial population structure among the DBLα types or microsatellite alleles. We tested for the existence of nonrandom structure in how the microsatellite alleles and DBLα types are organized into different hierarchical levels of geospatial location, all the way up from the level of the haplotype to that of the two catchment areas. A simple randomization of microsatellite alleles in the two catchment areas (*N* = 10,000) reveals that the observed number of shared microsatellite alleles between the two areas, which is 98, is not significantly different from the random expectation. The *p*‐value for this observed number of shared alleles is 0.59, reflecting that it is right in line with the random expectation (Figure 4a). Thus, there is no difference in microsatellite allele diversity between the two catchment areas. These findings are consistent with a detailed analysis of microsatellite data for this region in Ruybal‐Pesantez et al. (2017). We also tested for signals of geospatial structure in the DBLα type diversity using population genetic approaches and randomizations that rely on the predefined geographical divisions. Based on the randomizations, it seems that the main difference between the catchment areas with respect to DBLα type diversity is a modest difference in the diversity levels, as opposed to any clear differentiation in the identity of the types present in the two samples (Figure 4b and see additional discussion of the results in Supplementary Materials). We also found that *var* expected homozygosity (1 − *H_v_*) does not significantly differ between the two catchment areas (A and B), or between either of the catchment areas and the whole population. *Var* expected homozygosity (1 − *H_v_*) does, however, have a much broader distribution within the Vea/Gowrie catchment area and within the Soe catchment area than in the combined population (Figure 5b and see additional discussion of the results Supplementary Materials). Furthermore, 1 − *H_v_* has a much narrower distribution when pairs of DBLα types are taken from the whole population as opposed to one of the four villages or eight sections (data not shown). Our interpretation is that the difference between the expected homozygosity distributions for the whole population versus either of the catchment areas merely reflects differences in sample size. Our findings for *F*_*STv*_ among *var* genes in the different areas, villages, and sections are consistent with our other findings of little to no classic geospatial population structure (data not shown). In summary, sampling location does not appear to be a confounding variable because we found little to no classic geospatial population structure among the DBLα types or microsatellite alleles.

**Figure 6:**
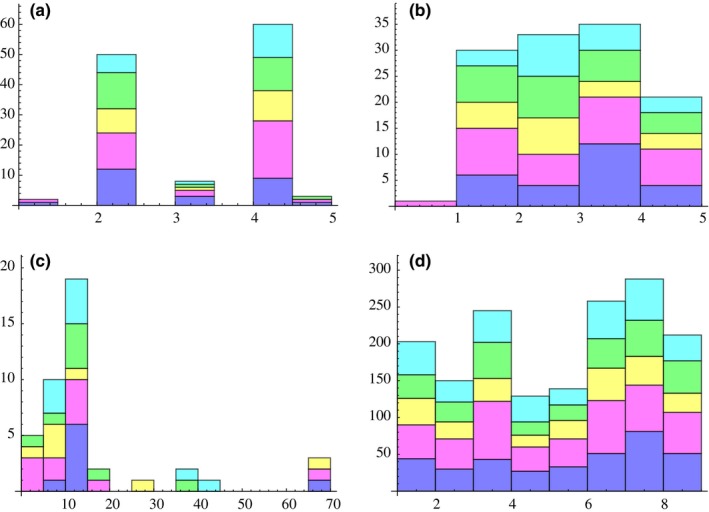
Potential factors explaining DBLα type modules. Purple = Module 1, Pink = Module 2, Yellow = Module 3, Green = Module 4, Turquoise = Module 5. No association apparent between module membership and the number of cysteines, MOI, sampling location or host age. (a) Number of cysteine residues within the DBLα tag region for a given type, and types grouped by module. (b) Average MSP2‐estimated MOI for a given type, and types grouped by module. (c) Host age for a given type, and types grouped by module. (d) Sampling location(s) for a given type and types grouped by module. Vea Gonga = 1; Vea Nayire = 2; Gowrie Nayire ‐ Kura = 3; Gowrie Tingre = 4; Soe Boko Mission Area = 5; Soe Boko Tamoliga = 6; Soe Sanabisi Tindingo = 7; Soe Sanabisi Akulgoo = 8

**Figure 7:**
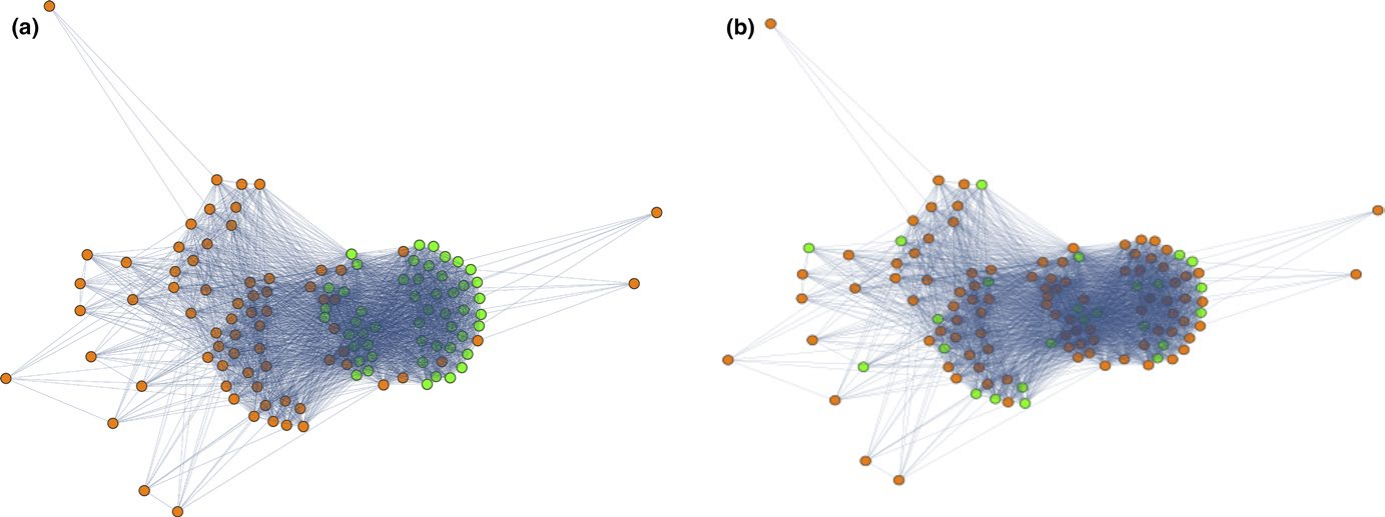
(a) The HB sharing network of DBLα types, for HBs with less than 50% frequency. The clustering of Cys2 *var* genes, shown in green (remaining nodes in orange), demonstrate that the HB sharing network is descriptive of *var* recombination constraints. (b) The *var* linkage modules cannot be explained by the structure of the recombination network because the *var* linkage modules do not cluster on the recombination network. Module 1 *var* genes are shown in green (remaining nodes in orange); the other modules also do not cluster (data not shown).

We asked next whether other variables such as host age, host MOI, or the *var* recombination hierarchy could explain the five *var* modules. We first addressed whether the *var* linkage modules were the result of recombination preferentially occurring within certain classes of *var* genes (i.e., the so‐called *var* recombination hierarchy). We did this by considering the number of cysteines in the DBLα tag—a feature that is highly conserved in different functional/recombination groups of DBLα sequence types. We found that cys2 DBLα classification did not correlate at all with module membership (Figure 6a), indicating that the modules are not the result of cys2 preferential recombination. We also tested whether the modules could be explained by a recombination hierarchy by building an HB recombination network and mapping cys2 sequences onto the recombination modules to confirm that these sequences clustered within the network, which they did (Figure 7a). We then mapped our modules onto this network and looked for clustering. We did not observe clustering for any of the modules (Figure 7b). These results indicate that the five modules in the DBLα type linkage network cannot be explained by the *var* recombination hierarchy and that they are not related to recombination mechanisms in any way.

We also considered whether the modules could be explained by multiplicity of infection (MOI) in the host, as it is conceivable that some DBLα types may only circulate in certain immune contexts. For example, if there is indeed a trade‐off between *Pf*EMP1 function and immune escape, then DBLα types that encode high‐affinity forms of *Pf*EMP1 may only circulate in relatively immunologically naïve hosts. However, we found no correlation between linkage module membership and the average multiplicity of infection of the hosts carrying a particular DBLα type (Figure 6b).

We considered whether the modules could be explained by host age, as it is conceivable that some DBLα types may circulate in only the youngest children, as the expression of some DBLα types has been associated with young age and/or certain age‐related disease manifestations. However, we found no correlation between linkage module membership and the average age of the hosts carrying a particular DBLα type either (Figure 6c).

In order to readdress whether sampling location could explain the modules, we also directly tested for whether there was any correlation between the sampling location(s) in which we observed certain DBLα types and module membership. This analysis was carried out in an analogous manner to how we tested for a correlation between module membership and host MOI or age. Again, we found no correlation between sampling location and module membership (Figure 6d).

**Figure 8:**
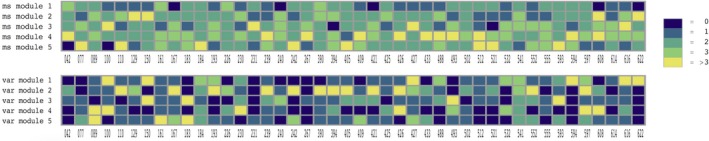
Comparison of the five *var* and five microsatellite modules by considering the contribution of each of these modules to each of the isolates for which there is both *var* and microsatellite data. Based on this analysis, the *var* and microsatellite modules appear to be unrelated

Finally, we wanted to compare the *var* linkage modules to another biological null hypothesis: the microsatellite linkage modules. The microsatellites should capture any neutral genomic structure resulting from demographic processes or mechanical genetic processes affecting the entire genome. We addressed whether there are modules in the microsatellite allele linkage network, and if so, whether they correlate with the DBLα type linkage modules to some extent. We built a microsatellite allele linkage network in the same way as we built a DBLα type linkage network, but instead of using the 29 single infections, we used the 55 isolates for which we had a high confidence complete set of microsatellite alleles. The software program MODULAR found five modules in the microsatellite allele linkage network. These five modules do not appear to be at all related to the five DBLα type linkage modules because when we compared the pattern of isolates within modules for these two different linkage networks, we did not observe any correspondence or similarity between any of the modules in the two different networks (Figure 8).

## 4 DISCUSSION

We observed a significant nonrandom structure in the DBLα type diversity within our sampling area. DBLα types do not travel through the host population randomly and independently. Specifically, *var* DBLα types appear to travel together in five loose linkage modules, with a clique of three DBLα types traveling together in an extremely conserved manner. Moreover, neither the five modules nor the clique appear to be the result of a *var* recombination hierarchy. Nor do they associate with a particular sampling location or host age or multiplicity of infection. Furthermore, the five modules and the conserved clique appear to be shaped by forces other than the demographic forces shaping diversity in the rest of the genome, as their occurrence is not correlated with microsatellite allele modules or microsatellite haplotype relatedness. Thus, the *var* linkage modules are likely shaped by more than just the neutral forces that act on the genome as a whole. In particular, it is possible that the five DBLα type linkage modules reflect a strain structure possibly caused by competition for host immune space, as predicted by theory (Artzy‐Randrup et al., 2012), and supported by serological observations before the *var* genes were discovered (Gupta, Trenholme, Anderson, & Day, 1994), and by serological networks of cross‐protection (Buckee, Bull, & Gupta, 2009).

The microsatellite diversity of this population is extensive and described in greater detail in another study (Ruybal‐Pesantez et al., 2017). In fact, all combinations of the 12 microsatellite alleles (haplotypes) within the estimated dominant infections and high confidence infections were unique. Despite this, some linkage structure in the microsatellite diversity was apparent. The index of association in the total sample and in subsamples was found to be statistically significant for all dominant infections and for the subset of high confidence infections (Ruybal‐Pesantez et al., 2017), both within catchment areas and villages. The population is nevertheless well mixed with regard to sampling location, host age‐group, and MOI, as these factors do not appear to explain or correlate with the observed linkage disequilibrium (using both PCA and model‐based analyses) (Ruybal‐Pesántez, 2016; Ruybal‐Pesantez et al., 2017). Therefore, it is our interpretation that microsatellite linkage disequilibrium does not result from a complex constraint on the population structure. Rather, it is likely a reflection of the fact that we sampled a high proportion of the host population at the end of the dry season, when MOI is sufficiently low (median of 2) ‐ conditions that may promote selfing within the parasite population. In simple terms, the linkage we observe likely reflects the fact that the parasite population is finite in size.

We also did not observe strong differentiation in the *var* diversity between the two catchment areas—the main difference being a modest difference in the diversity levels between the samples from the two areas (see Supplementary Materials). However, given the extensive diversity of the microsatellite loci sampled, further sampling of this host population may reveal patterns of *var* structure not yet observable in this study.

We found that field isolates are on average less related than if they were random subsets of the *var* diversity, but that simultaneously there also exists rare pairs of highly related isolates, which are also not expected randomly. We attribute the nonrandom attributes of the distribution of DBLα types in isolates to a combination of the complex transmission dynamics that mediate the opportunities for recombination among *var* repertoires, and a selective response to avoid competition with cocirculating parasites niche partitioning. The nonrandomly low median is consistent with the outcome of selection for minimal antigenic overlap among parasites sharing a common host resource, while the existence of highly related pairs of isolates, and thus a high observed maximum overlap among isolates, is likely due to the unavoidable relatedness of some pairs of parasites in any deeply sampled, finite transmission system. Our interpretation is based on our observation of nonrandom patterns among DBLα types and isolates repertoire overlap indices, along with our failure to observe possible confounding variables: geospatial population structure or other forms of complex population/transmission structure among the microsatellite alleles or the DBLα types.

The clique of three highly linked DBLα types did not appear to be related to sampling location or background relatedness as described by microsatellites, as isolates that contain the clique have lower than average relatedness by microsatellites. The clique is therefore an interesting antigenic module that could be studied in more detail in the future.

We did not detect classic geospatial population structure in the DBLα type diversity even at a fine scale (<10 km). Our results contrast with recent population genetic results for *var* genes in another endemic area, in Papua New Guinea (PNG) (Tessema et al., 2015), where the authors found *var* type diversity to reflect fine‐scale geospatial location of their samples even when microsatellite markers did not reveal population structure. Our finding of no geospatial population structure among DBLα type diversity may reflect the order of magnitude greater *var* diversity in many African villages as compared to areas in PNG.

The lack of classic geospatial population structure among the DBLα type diversity is not merely a reflection of high diversity, however. While we observe relatively few DBLα types more than once, we nevertheless sample a sufficient number multiple times in order to describe strong geospatial population structure if it existed. We can determine that repeated DBLα types do not occur within the same sampling location more than expected at random. Therefore, these populations are not simply diverse with respect to their DBLα types, they are diverse and *well mixed*, and this is at all geospatial scales analyzed in this study. Higher resolution population genetic spatial analysis combined with deeper sampling of the pathogen population is warranted.

Prior research has suggested that a *var* recombination hierarchy may function to maintain and reinforce three *var* functional types (A, B, and C) (Kraemer et al., 2007). The importance of these three *var* functional types has received much attention, and much effort has gone into classifying all existing *var* diversity (with the exception of the three “strain transcendent” *vars*) into these three categories. It has been suggested that this recombination hierarchy is the primary force responsible for the current diversity of *var* genes globally. Other recent results, which emphasize the role of mitotic recombination in generating *var* diversity (Claessens et al., 2014), have made this picture more complete by giving a possible explanation for how new mosaic *var* genes can be generated in every parasite life cycle within an infected individual. Our findings suggest that this may not be the whole story, as *var* diversity has a transmission structure that is not explainable by this recombination hierarchy or by simple genomic or demographic forces shaping microsatellite diversity. It is possible that antigenic selection is playing an important role in shaping *var* diversity. The structure of *var* diversity would therefore not just be the result of recombination constraints in combination with demographic forces such as transmission dynamics, nonrandom mating, and drift.

We reason that selection may promote transmission of *var* genes in a module structure—especially in highly endemic, and thus competitive environments—for the sake of reducing competition by limiting similarity of cocirculating strains. Our findings on reduced overlap between *var* repertoires and the existence of modularity among *var* DBLα sequence types are consistent with a role for immune selection and the predictions of strain theory. Nevertheless, we believe further work is needed to go beyond statistical randomizations. Specifically, process‐based null models need to be considered to explicitly compare neutral and non‐neutral mechanisms, and strain theory itself requires extension to take into account the extremely high diversity of the system in terms of *var* type numbers and repertoire length, as well as functional differences between these types. Identifying the evolutionary and ecological dynamics that are responsible for the nonrandom structure in *P. falciparum* antigenic diversity is ultimately important for designing effective intervention in highly endemic areas.

## CONFLICT OF INTEREST

None declared.

## DATA ACCESSIBILITY

All sequence data used in this study have been submitted to GenBank: Project number PRJNA385207; Accession numbers SAMN06833123‐SAMN06833331.

## AUTHOR CONTRIBUTIONS

MMR designed and performed analyses and wrote the paper; YAR designed analyses in the manuscript and reviewed the manuscript; SRP contributed to laboratory work, sequenced the samples, and reviewed the manuscript; KET designed and contributed to data collection, laboratory work, sequencing of samples, and reviewed the manuscript; TSR contributed to sequencing and the initial sequence analysis to create the sequence database; AO, AG, and KK contributed to study design, coordination of field work, data collection, and reviewed the manuscript; KPD and MP conceived of the study, contributed to the design of analyses in the manuscript, and contributed to the writing of the manuscript.

## APPENDIX: INFORMATION BOX

### BACKGROUND ON *P. FALCIPARUM* AND *VAR* DIVERSITY

*P. falciparum* ‐ like parasites have been circulating in primates for at least 6–10 million years (Prugnolle et al., 2011) and *P. falciparum* is well adapted to its human host (Loy et al., 2016). The vast majority of infections do not even cause disease symptoms (>99%), and despite decades of intensive control efforts, *P. falciparum* continues to thrive within human populations worldwide, causing over 200 million malaria cases per year worldwide (WHO, 2017). Likewise, the human immune system is well adapted to this parasite. Human IgG responses are “exquisitely specific” to *falciparum* proteins (Fairhurst, 2015). Moreover, humans develop highly protective antidisease immunity to *P. falciparum* after only a few infections, which typically occur within the first few years of life in highly endemic areas (Marsh, 1992). Despite the ancientness of this coevolutionary relationship, the *falciparum* parasite is responsible for nearly all deaths due to malaria, which are currently estimated at 445000 annually, and which primarily occur in young children and primigravid women in Sub‐Saharan Africa (WHO, 2017). The mortality caused by this parasite is thought to be a consequence of a singular trait unique to the *falciparum* lineage: the infected erythrocytes massively sequester within host microvasculature (Smith, Rowe, Higgins, & Lavstsen, 2013).

A single parasite protein—*P. falciparum* erythrocyte membrane protein (*Pf*EMP1)—appears to underlie this unique virulence trait. This variant surface antigen is a large and sticky protein that is expressed on the surface of infected erythrocytes, where it binds host endothelial receptors, adhering infected erythrocytes to host microvasculature. Shape and rigidity changes in infected erythrocytes result in mechanical clearance if they circulate to the spleen, so *Pf*EMP1‐mediated sequestration in host tissues is critical for parasite survival. Abundant and exposed at the host–parasite interface, *Pf*EMP1 also appears to be the most important target of the human immune response (Chan, Fowkes, & Beeson, 2014). Although sterilizing immunity never develops against *P. falciparum*, immunity to disease typically follows from only one or two severe infections, and this process appears to centrally involve an immunoglobulin G (IgG) response to *Pf*EMP1, which prevents *Pf*EMP1 adhesion (Bull et al., 1998; Cham et al., 2009; Gupta, Snow, Donnelly, Marsh, & Newbold, 1999; Lusingu et al., 2006; Nielsen et al., 2002). *Pf*EMP1 is thus under selection to simultaneously bind host endothelial receptors and evade specific immunity (Lau et al., 2015). In response to this, the parasite encodes about 60 antigenically distinct *Pf*EMP1 variants per genome, and through the course of an infection it switches expression between these variants (Amit‐Avraham et al., 2015). Similar immune evasion mechanisms—so‐called “antigenic variation” systems—have evolved independently in several other parasite lineages (Deitsch et al., 2009).

The genes that encode *Pf*EMP1 variants are known as the *var* gene family, and they are one of the best‐studied classes of variant surface antigens. These genes are located in multiple subtelomeric and centromeric locations within the genome (Kyes, Kraemer, & Smith, 2007). Soon after the parasite invades a erythrocyte, uncharacterized gene regulation machinery allows only one *var* promoter to be active at a time (Frank & Deitsch, 2006; Voss et al., 2006). Antigenic variation explains the pathogen's remarkably long duration of infection under natural conditions (up to at least 250 days; Miller, Good, & Milon, 1994). Distinct *Pf*EMP1 variants come in a diversity of architectural types, and within‐domain amino acid identity is less than 50% even for *vars* of the same architectural type (Kraemer et al., 2007).

It has not been possible to address *var* sequence diversity in most studies of *P. falciparum* genome‐wide diversity (Jeffares et al., 2007; Kidgell et al., 2006; Osley & Shen, 2006; Volkman et al., 2007). With the exception of the *var* sequences within a few sequenced *P. falciparum*/*reichenowi* genomes, studies of *var* diversity have relied on sequencing from field samples using degenerate primers (Barry et al., 2007). These studies have established that—in addition to within‐genome *var* diversity—there is also immense diversity between the *var* repertoires of distinct parasites. Thousands of distinct *var* genes exist even within small local populations (Chen et al., 2011; Day et al., 2017). This extreme population‐level diversity is likely why sterilizing immunity fails to develop against this pathogen even after repeat infections, and why prevalence and multiplicity of infection can reach high levels in some areas.

*Var* diversity not only encodes antigenic variation, but also functional variation. With the exception of three highly conserved “strain transcendent” *var* types, *var* sequences can be classified into three groups based on upstream sequence (ups) and chromosomal location: groups A, B, and C (Smith, 2014), and to some extent, these correlate with the different architectural types. Sequence classification systems based on the presence of conserved domain cassettes, short specific sequence motifs, or homology blocks within *Pf*EMP1 coding sequence can further separate *var* genes into a larger number of categories, many of which have unique cytoadhesion traits implicated in severe disease, and/or are preferentially expressed in patients with severe disease symptoms (e.g., Rorick, Rask, Baskerville, Day, & Pascual, 2013).

The current model is that different *Pf*EMP1 variants exhibit tropism for different host microvascular beds, which in turn results in different manifestations of disease. Group A *var* genes tend to be expressed in patients with severe malaria, and other types of *vars* encode *Pf*EMP1 variants with especially high affinity for certain host endothelial receptors or cell types (Smith, 2014). Moreover, it appears likely that some of these type‐specific *Pf*EMP1 affinities play a role in adhesion‐based disease complications (e.g., Avril, Brazier, Melcher, Sampath, & Smith, 2013; Avril et al., 2012; Moxon et al., 2013). The relationships between specific *var* sequences and binding preferences in many cases remain consistent across global isolates. This indicates that these functional specializations arose early in the evolution of *P. falciparum* and that they have been maintained by selection (Smith, 2014).

*Var* sequence diversity is generated by ectopic recombination within the largely nonallelic gene family and primarily results in gene conversion events (Kyes et al., 2007). It is thought that the mechanism involves the formation of “bouquets” where subtelomeric and centromeric *var* genes come together physically at the nuclear periphery. It appears that successful recombination events preferentially occur within the A/B/C *var* gene groups, possibly in order to maintain the functional specializations and/or allowing for functional divergence (Smith, 2014). The asymmetry in recombination rates within versus between different *var* groups is referred to as the “recombination hierarchy”. The gene regulatory mechanisms responsible for *var* gene expression switching and control may be overlapping with *var* recombination mechanism, but whether the activation of *var* gene expression actually induces mitotic recombination events remains unknown (Kyes et al., 2007). Whether diversity is generated primarily through meiotic or mitotic recombination also remains an active topic of debate (Claessens et al., 2014).
