# Supplementary material for: Signatures of competition and strain structure within the major blood‐stage antigen of Plasmodium falciparum in a local community in Ghana
Source: Ecol Evol. 2018 Mar 1;8(7):3574–88. doi: 10.1002/ece3.3803 (PMC5901166; doi:10.1002/ece3.3803)
Supplement: Supplementary file 1 [file ECE3-8-3574-s001.pdf]

Additional results

MSP2 versus Microsatellite to estimate MOI:
We collected MSP2 information for all 209 isolates in the *var* dataset, however we used microsatellite alleles to estimate MOI because it is more conservative. Microsatellite maximum allele count gives an average MOI of 2.97 versus 2.06 using MSP2 for the same 196 isolates.

Number of *var* types shared between areas:
We considered whether the number of *var* types shared between the two areas was greater or smaller than the random expectation, based on 10,000 randomizations (Figure 5b). We find 6095 *var* types in Vea/Gowrie, 7128 *var* types in Soe, and 1915 *var* types that are common to both areas. By performing 10,000 randomizations, we find that this is a significantly smaller number of shared *var* types between the two regions than expected at random (p=0.0179 for this value or lower). However this may merely reflect a difference in diversity between the two areas. Vea/Gowrie is considerably less diverse than Soe despite having essentially the same sample size. Because the difference in diversity between the areas is not preserved in the randomizations, it could explain the smaller-than-expected number of shared types between the two areas. In summary, the main difference between the samples from the two areas with respect to *var* type diversity is just a modest difference in the diversity levels, as opposed to any clear differentiation in the identity of the types present in the two samples. It is worth noting that the difference in diversity is a type of population structure nonetheless, and that it is not reflected by microsatellites when we apply the same tests to them. The same microsatellite alleles, distributed in the same way, are found in both catchment areas.

Expected homozygosity:
We tested for geographic population structure within the *var* sample using a number of addition methods related to traditional population genetics statistics. In the Methods section we describe how these are interpreted in this unusual genetic context, and we define unique variations of traditional population genetic statistics: e.g., *var* expected heterozygosity (Hv). We asked whether the expected homozygosity within Vea/Gowrie, or within Soe, is significantly greater than the expected homozygosity within the combined catchment areas. In other words, are parasites made exclusively from variants in Vea/Gowrie or the variants made exclusively from variants in Soe more homozygous than parasites made from a random combination of genes from both catchment areas? We find that *var* expected homozygosity (1-Hv) does not significantly differ between the two catchment areas, or between either of the catchment areas and the whole population. However, *var* expected homozygosity (1-Hv) has a much broader distribution within Vea/Gowrie and within Soe than in the combined population (Figure 6B). Furthermore, 1-Hv has a much narrower distribution when pairs of *var* types are taken from the whole population as opposed to one of the four sub-populations or eight villages (data not shown). In summary, our interpretation is that the difference between the expected homozygosity distribution for the whole population versus either of the catchment

areas likely reflects differences in sample size for the entire sample compared to either of the catchment areas on its own. Our findings for Fstv among *var* genes in the different areas, sub-populations, and villages are consistent with our other findings of little to no classic geospatial population structure (data not shown).
